# Proceedings of the Society of Electro-Therapeutists

**Published:** 1894-11

**Authors:** Clara E. Gary

**Affiliations:** Secretary


### PROCEEDINGS OF THE SOCIETY OF ELECTRO-THERAPEUTISTS.

The National Society of Electro-Therapeutists held its second annual meeting in Berkeley Lyceum, New York city, September 20th and 21st.

President William Harvey King, M. D., called the members to order, and made an able address, earnestly impressing upon the minds of his audience the necessity of electro-therapeutic teaching.

The following papers were read:

"An Investigation of Interpolar Action in Galvanic Currents," by William L. Jackson, M. D., Boston, Mass.

"Investigations Regarding the Use of Static Electricity," by Frank E. Caldwell, M. D., Brooklyn, N. Y.

"Some Observations on the Influence of Electricity on Muscular Development," by William H. King, M. D., New York city.

"Use of Electricity in Orificial Surgery," by C. A. Weirick, M. D., Chicago, Ill.

"Clinical Use of Electricity in Muscular Development," by William H. King, M. D., New York city.

"Radical Electrolysis," by F. M. Frazer, M. D., New York city.

"Hints on the Use of Electricity in Gynæcology," by Flora A. Brewster, M. D., Baltimore, Md.

"My Experience in Regard to the Susceptibility to the Electrical Current," by Jennie W. Martine, M. D., New York city.

"The Physical Properties of the Sinusoidal Current," by J. W. Gladstone, M. D., New York city.

"The Exhibition of an Apparatus for the Application of Heated Oxygen on Ozenized Oxygen by Electrical Propulsion," by Irving Townsend, M. D., New York city.

"Exhibition of an Electrical Endoscope, Laryngoscope, and Stethoscope," by M. Milton Weill, M. D., New York city.

"Diphtheritic Paralysis Treated by Electricity," by William L. Jackson, M. D., Boston, Mass.

"Brief Researches on the Action of Galvanic, Faradic, and Franklinic Currents on Nervous Tissue," by Walter Y. Cowl, M. D., Berlin, Germany. Presented by J. T. O'Connor, M. D., New York city.

"Electrical Massage in the Treatment of Diseases of the Ear," by Thomas L. Shearer," M. B. C. M., Baltimore, Md.

"The Galvanic Current of High Amperage in the Diseases of the Liver," by Lorenzo J. Kohnstamm, M. D., New York city.

"Details in the Instrumentation in Diseases of the Ear," by Henry C. Houghton, M. D., New York city.

"Report of Clinical Cases," by M. Bonner Flynn, M. D., Worcester, Mass.

"A New Method of Treatment for Gouty Arthritis of the Fingers," by Frank A. Gardner, M. D., Washington, D. C.

"Electricity as a Therapeutic Means at River View Home," by W. S. Watson, M. D., Fishkill, N. Y.

"Inter-Uterine Cataphoresis," by William H. King, M. D., New York city.

"Cicatrix of the Cervix Uteri Treated by the Negative Pole of the Galvanic Battery," by Alice B. Condict, M. D., Orange, N. J.

"The Electrical Treatment of Appendicitis," by W. N. Williams, M. D., San José, Cal.

"New Painless Method of Removing Facial Blemishes by Electrolysis," by H. E. Waite, M. D., New York city.

"An Electric Potpourri," by A. S. Bailey, M. D., Atlantic City, N. J.

"Electric Induction Cabinet and Some of its Uses," by J. H. Woodward, M. D., Seward, Neb.

"How to Measure the Faradic Current," by Harry F. Waite, New York city.

"Clinical Reports of Electricity in Gynæcology," by Bessie F. Haines, M. D., St. Paul, Minn.

The papers read were intensely interesting and suggestive.

Many of them were the product of original research, and the discussions following their reading were very instructive.

The Society owes to its retiring President, Dr. W. H. King, not only its existence, but most of the success and value of the meetings.

The following officers were elected for the ensuing year: President, William L. Jackson, M. D., Boston, Mass.; First Vice-President, E. S. Bailey, M. D., Chicago, Ill.; Second Vice-President, F. A. Gardner, M. D., Washington, D. C.; Treasurer, J. B. Garrison, M. D., New York city; Secretary, Clara E. Gary, M. D., Boston, Mass.

W. H. King, M. D., New York city, and M. D. Youngman, M. D., of Atlantic City, were elected to act with the officers as an Executive Committee.

The Society adjourned to meet next September in Boston.

Clara E. Gary, M. D., *Secretary.*

